# Relatedness, Conflict, and the Evolution of Eusociality

**DOI:** 10.1371/journal.pbio.1002098

**Published:** 2015-03-23

**Authors:** Xiaoyun Liao, Stephen Rong, David C. Queller

**Affiliations:** 1 Department of Ecology and Evolutionary Biology, Rice University, Houston, Texas, United States of America; 2 Biology Department, Washington University in St. Louis, St. Louis, Missouri, United States of America; University of Lausanne, SWITZERLAND

## Abstract

The evolution of sterile worker castes in eusocial insects was a major problem in evolutionary theory until Hamilton developed a method called inclusive fitness. He used it to show that sterile castes could evolve via kin selection, in which a gene for altruistic sterility is favored when the altruism sufficiently benefits relatives carrying the gene. Inclusive fitness theory is well supported empirically and has been applied to many other areas, but a recent paper argued that the general method of inclusive fitness was wrong and advocated an alternative population genetic method. The claim of these authors was bolstered by a new model of the evolution of eusociality with novel conclusions that appeared to overturn some major results from inclusive fitness. Here we report an expanded examination of this kind of model for the evolution of eusociality and show that all three of its apparently novel conclusions are essentially false. Contrary to their claims, genetic relatedness is important and causal, workers are agents that can evolve to be in conflict with the queen, and eusociality is not so difficult to evolve. The misleading conclusions all resulted not from incorrect math but from overgeneralizing from narrow assumptions or parameter values. For example, all of their models implicitly assumed high relatedness, but modifying the model to allow lower relatedness shows that relatedness is essential and causal in the evolution of eusociality. Their modeling strategy, properly applied, actually confirms major insights of inclusive fitness studies of kin selection. This broad agreement of different models shows that social evolution theory, rather than being in turmoil, is supported by multiple theoretical approaches. It also suggests that extensive prior work using inclusive fitness, from microbial interactions to human evolution, should be considered robust unless shown otherwise.

## Abstract 2

Mathematical modelling shows that the evolution of sterile castes requires genetic relatedness but also involves conflicts between kin; these results contradict recent claims but agree with inclusive fitness theory.

## Author Summary

The evolution of sterile worker castes in social insects has fascinated biologists ever since Darwin; how can selection favor a trait that decreases reproductive fitness? W. D. Hamilton solved this dilemma in the 1960s with a theory showing that reproductive altruism could evolve if it increased the worker’s inclusive fitness, which included effects that it had on increasing the fitness of its relatives. This solution to a crucial evolutionary problem, sometimes called kin selection, was challenged in a recent paper. The paper generated much controversy, but no one has contested its new theoretical model of the evolution of eusociality, which appeared to overturn much of what was previously thought to be true from kin selection theory. Here we examine this model in greater depth, showing that its apparently novel conclusions are overgeneralized from narrow and often inappropriate assumptions. Instead, this modeling strategy yields results that confirm important insights from kin selection and inclusive fitness, such as the importance of relatedness and the existence of conflicts in social insect colonies.

## Introduction

The eusocial insects have occupied an important place in biology because of their extraordinary levels of cooperation [1–4]. In ants, termites, some bees, some wasps, and a few other taxa, certain individuals, called workers, give up their own reproduction in order to help others reproduce. Darwin was vexed over the question of how such reproductive altruism evolves or indeed how any traits of sterile workers evolve, but he believed that such sterility was due to some form of selection at the family level or at the group level [5]. Hamilton provided the first rigorous treatment of this idea, with a key insight being the importance of genetic relatedness [1]. A conditional gene causing a worker to give up reproduction could be favored if it provided sufficient help to a relative who would share that gene at above-random levels. He showed that this process, which became known as kin selection, could be analyzed by summing up an actor’s fitness effects, each multiplied by the actor’s relatedness to the individual receiving the fitness effect. When this sum, called the inclusive fitness effect, is positive, the trait should be favored by selection. For giving up one’s reproduction (fitness cost *c*) to benefit other individuals (total fitness gain *b*) related by *r*, the inclusive fitness condition is −*c* + *rb* > 0.

Kin selection and inclusive fitness became the dominant modes of thinking about the evolution of eusocial insects [4,6,7], and their success in this area has led to them being applied to many other problems in social evolution [8–12]. Recently, this paradigm was criticized by Nowak et al. [13], who argued that inclusive fitness was an inaccurate and unnecessary method and that kin selection was not a very useful way to think about social evolution. Both of these conclusions have in turn been extensively criticized as depending on multiple misconceptions [14–22]. We concur with many of these criticisms but do not revisit them here. Instead, we offer a different kind of critique of the Nowak et al. paper. To provide an example that bolstered their general arguments, Nowak et al. [13] also developed their own mathematical model of the evolution of eusociality, presenting it as an example of a modeling approach that is superior to inclusive fitness modeling. However, as has been recently pointed out [23], this eusociality model has scarcely been addressed.

We do not contest this modeling approach. Instead, we accept it as valid and use it to show that its implementation in Nowak et al. [13] led to errors of interpretation that greatly overstated any differences with standard inclusive fitness results. We do not address the exact quantitative match of the two approaches but instead focus on large apparent discrepancies of interest to empiricists. Because their model is claimed to be superior to inclusive fitness, we focus on three of their conclusions that seem at greatest variance with the conventional inclusive fitness and kin selection view of the evolution of eusociality. In each case, we will show that the kin selection view is essentially confirmed. Nowak et al. [13] also make other assertions about eusociality that are consistent with inclusive fitness theory, such as the importance of grouping and preadaptations. We ignore these in order to focus on the seemingly novel conclusions of the Nowak et al. model. The first two of these are fundamental qualitative differences from inclusive fitness, while the last is more a difference in degree.

First, Nowak et al. [13], following earlier work by Wilson [24,25], claimed that relatedness was not an essential element in the evolution of eusociality. They wrote that “relatedness is better explained as a consequence rather than as the cause of sociality,” that “grouping by family hastens the spread of eusocial alleles but it is not a causative agent,” and that “relatedness does not drive the evolution of eusociality” [13]. In the same vein, they also contest empirical evidence that relatedness is important [13]. We take causality to mean that variation in relatedness leads to variation in the likelihood of evolving eusociality. As has previously been pointed out, the Nowak et al. model could not test this because it was based on groups of relatives, with no comparable model of unrelated individuals being presented [15,20]. Nowak et al. appear to have partially accepted this point: “One, we do not argue that relatedness is unimportant. Relatedness is an aspect of population structure, which affects evolution” [26]. However, this response leaves unanswered exactly how it affects evolution. At least one of the authors [27] continues to assert that relatedness only hastens the spread of alleles and that it is not causal. To test these claims, we extend their model to cases in which relatedness can vary.

Second, whereas inclusive fitness theory has emphasized that cooperation occurs in the face of potential and actual conflicts among colony members with different interests [4,7,28,29], Nowak et al. [13] assert that the colony as a whole is all that matters. They argue that “the workers are not independent agents,” that “their properties are determined by the alleles that are present in the queen (both in her own genome and in that of the sperm she has stored),” that “the workers can be seen as ‘robots’ that are built by the queen,” and that they “are part of the queen’s strategy for reproduction” [13]. Nor, contrary to earlier work by Wilson [24,25], do they brook any conflicts between levels of selection: “there is only one level of selection, the hymenopteran colony, which is treated as an extension of the queen, whose genes are the units of selection” [13]. To test whether workers and queens are independent agents that are selected differently, we construct parallel models in which the genes determining whether their offspring stay and help are expressed in mothers or expressed in offspring.

Finally, Nowak et al. [13] claim that eusociality is harder to evolve than has been appreciated. They write that “a key observation of our model is that it is difficult to evolve eusociality, because we need very favorable parameters” and that “despite the obvious and intuitive advantages of eusociality, it is very hard for a solitary species to achieve it” [13]. If there is any novelty in this conclusion, it must be that eusociality is harder to evolve than has been thought previously; that is, it is harder to evolve than predicted from inclusive fitness effects (–*c* + *rb* > 0). We explore how this conclusion changes with reasonable alterations in the fitness functions and the worker decision rules.

If the three apparently novel conclusions of Nowak et al. are correct [13], then inclusive fitness theory could be said to have made some serious errors, and we might have to throw out or rethink important elements of the last 50 years of social evolution theory. If instead our models reject those apparently novel conclusions in favor of results consistent with those obtained through inclusive fitness, it would show that different theoretical approaches yield broadly consistent results, as they ought to in a healthy science.

## Results

We modify the Nowak et al. [13] haploid model, which is simpler than their haplodiploid one but sufficient to demonstrate the important points. Our goal is not to exactly model eusociality in any particular organism but to examine the logic and truth of three general claims in Nowak et al. [13], claims that pertain to both the haploid and haplodiploid models. The basic model includes solitary and eusocial genotypes expressed in offspring, where solitaries always leave to reproduce, while eusocials stay and help their mother with probability *q* and leave to reproduce with probability 1 – *q*. Mothers and offspring are genetically identical. Differential equations describe changes in the numbers of solitary individuals and eusocial colonies based on colony-size–specific queen birthrates (*b*_*i*_) and death rates (*d*_*i*_), as well as worker death rates (*α*) and density dependence (*η*) (see Methods, Equation 1). If larger colony size (more workers) sufficiently increases the queen’s birthrate and/or decreases her death rate, the eusocial type can be favored over solitary reproduction under some probabilities of staying *q*. Using these equations, we recovered results indistinguishable from those of Nowak et al. [13] (e.g., their Figure 4). We then explored the effects of various assumptions by changing them one by one.

First, the models of Nowak et al. [13] assumed eusocial offspring stay with their mother so that there was always genetic relatedness among participants. In the haploid model, this meant that helpers were genetically identical (*r* = 1) to their mother and to the siblings they raised. To vary genetic relatedness in the haploid model, we allowed some offspring mixing between mothers before implementing their genetic helping rules. Each offspring has a probability *r* of being with her own mother before deciding whether to help her or leave to reproduce and a probability 1 – *r* of being with a random mother. This could result from offspring movement between nests, from mothers laying a fraction of their eggs in other nests, or from nest usurpation [30,31]. *r* is equivalent to relatedness to the new mother (after movement) because it represents identity to that mother above chance levels; a fraction *r* is identical to the head of their colony and her offspring (*r* = 1), while the remainder are randomly associated with colonies (*r* = 0). After this temporary mixing, offspring execute the original Nowak et al. strategies: offspring with the solitary genotype always leave to reproduce alone, and offspring with the eusocial genotype stay and help their colony with probability *q*. Differential equations implementing this model are given in the Methods (Equation 2).

**Fig 1:**
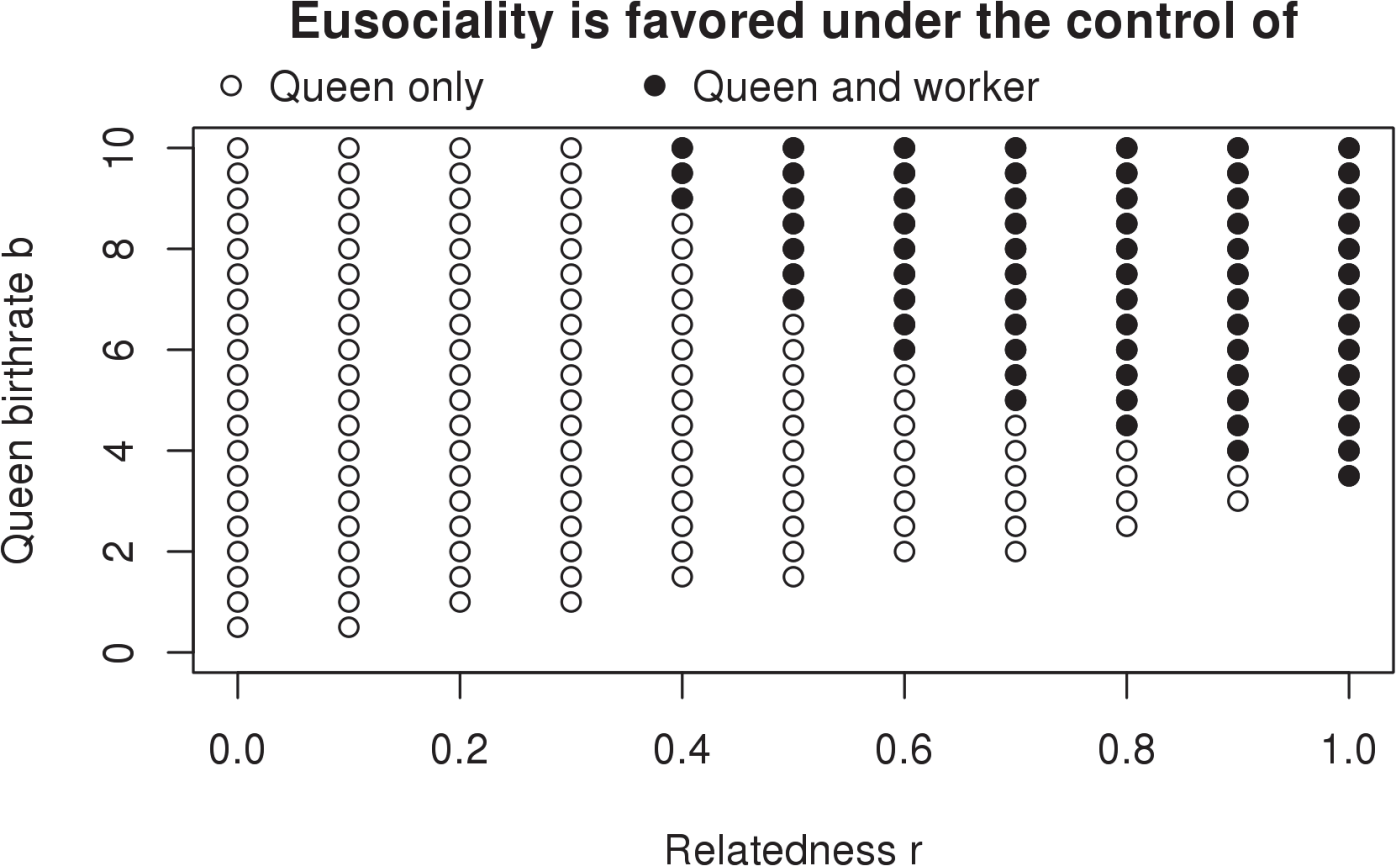
Relatedness (*r*) and the evolution of eusociality. The worker-assisted birthrate *b* and the probability of staying *q* are allowed to vary, while other parameters are as in Figure 4 of Nowak et al. [13] (*m* = 3, *b*_*0*_ = 0.5, *d*_*0*_ = 0.1, *d* = 0.01, α = 0.1, η = 0.01). Filled circles show values of relatedness *r* and worker-assisted queen birthrate *b* that select for eusociality (for at least one value of *q*) if the decision is made by offspring (Equation 2). Reducing relatedness makes eusociality harder to evolve (requires higher *b*). When the decision is made by genes acting in mothers (Equation 3), eusociality evolves under much broader conditions (open and filled circles), and lowering relatedness make eusociality easier to evolve. The open circles represent the zone of potential conflict, in which mothers but not offspring favor eusociality. The data used to make this figure can be found in S1 Dataset.

The filled circles in Fig. 1 show when selection on offspring favors eusociality under varying relatedness *r*, worker-assisted queen birthrate *b*, and probability of staying *q* (other parameters continue to match the standard Nowak et al. Figure 4 parameter values). Lowering relatedness clearly makes it more difficult for eusociality to evolve; with lower *r*, a higher *b* is required to favor eusociality. In the extreme, when offspring are randomly associated with colonies so that relatedness is zero, even *b* = 500 (a 1,000-fold increase in the queen’s birthrate due to helpers) is insufficient to favor eusociality. As expected from inclusive fitness theory, relatedness is causal in the sense that some relatedness is necessary for eusociality and increasing relatedness increases the range of conditions allowing eusociality to evolve.

Second, to address the issue of whether worker offspring are independent agents or simply robots carrying out the queen’s interests, we need to compare models of control by different agents. This means comparing models in which the decision to stay and help is made by genes in offspring bodies to models in which it is made by genes in the resident queens’ bodies. Though Nowak et al. [13] seem to argue for queen control, their models are for offspring control because they generally assume that genes expressed in worker bodies determine the decision to stay or leave.

However, inclusive fitness theory predicts that when queen control is possible, it will generally be more favorable for evolving eusociality [7] unless relatedness is one, in which case no conflict is expected. To model queen control under varying relatedness in the haploid model, we allowed offspring to mix exactly as in the offspring control model above but then allowed the resident queen’s genotype to determine if her mixed offspring pool helps or not. If the mother has the solitary genotype, all of her mixed pool disperses to become reproductives; if the mother has the eusocial genotype, she causes a fraction *q* of her offspring pool to stay and help her, independent of offspring genotype. Differential equations governing this system are given in the Methods (Equation 3). As predicted by inclusive fitness theory, eusociality evolves much more easily under queen control (Fig. 1, all circles). The only exception, as expected under inclusive fitness theory, is when there is no mixing between nests so *r* = 1 and the two decision rules are selected identically. In fact, assuming that queens can control the trait, we see the expected opposite relationship with relatedness; the less related the queen is to the offspring in her colony, the more the queen is selected to cause them to be workers.

The final claim that we examine is that eusociality is hard to evolve [13]. This depends on what is meant by “hard,” but we can usefully ask whether eusociality is as difficult to evolve as is implied in the Nowak et al. [13] paper. Their claim seems based on particular and odd choices for fitness functions and worker decision rules. The fitness function that they generally explored was a threshold function in which workers add no fitness gains to the queen below a colony of size *m* and add a fixed gain (increasing queen *b* or decreasing *d*) in colonies at or above size *m*, regardless of how many workers are added. This means that workers in colonies below that threshold contribute nothing until enough further workers join and that workers above the threshold also add nothing extra unless other workers die, returning the colony to the threshold. If most workers are contributing nothing, then it is not surprising that eusociality would be hard to evolve. In the example most explored, the threshold colony size *m* was set at 3 (their Figure 4), such that two workers were needed to raise the queen’s birthrate from *b*_*0*_ = 0.5 to *b* = 4 and to lower her death rate from *d*_*0*_ = 0.1 to *d* = 0.01 (they also let α = 0.1 and *η* = 0.01) [13]. This 8-fold increase in the queen’s birthrate allowed eusociality to evolve for some values of *q*, but lower values of *b* did not allow eusociality to evolve. Not surprisingly, requiring more workers before the queen increased fitness (higher *m* thresholds) made eusociality even more difficult to evolve.

As noted above, the assumption that workers must stay with probability *q*, regardless of the state of the colony, means they may be maladaptively staying in colonies that are too large to gain further benefits. It should be easy for workers to avoid this problem. For example, they might instead implement the rule to stay when the colony is below some threshold size *w* and leave when it is at or above that size. We implemented differential equations to model this change of assumption (see Methods, Equation 4) in the original Nowak et al. model with worker control and *r* = 1 (i.e., independently of the other changes explored above). Eusociality does evolve more readily. For example, for the same parameter values as in Figure 4 of Nowak et al., eusociality can now be favored under a somewhat lower benefits threshold (b = 3), that is, when helped queens get a 6-fold advantage.

**Fig 2:**
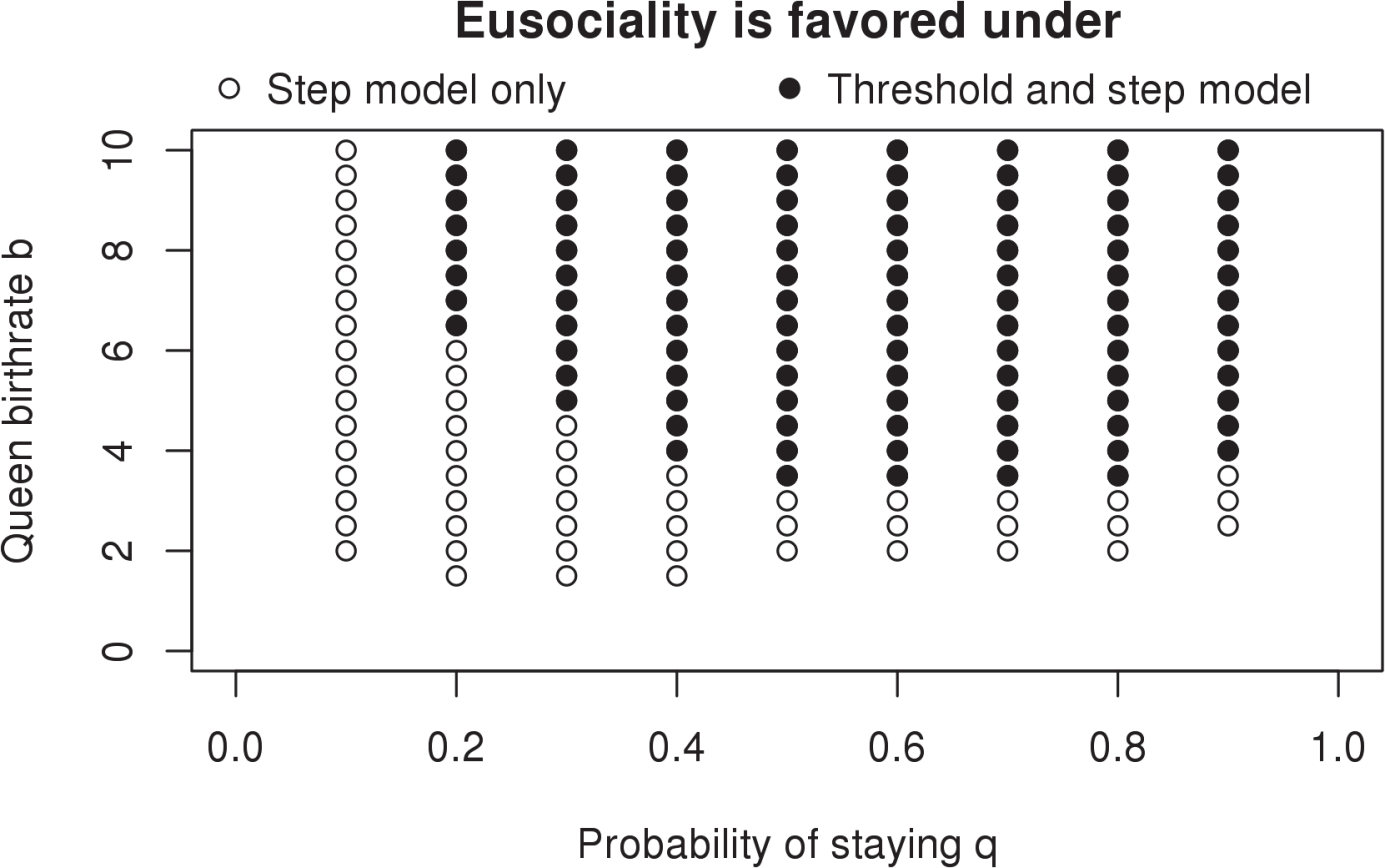
Eusociality evolves more readily under a step model (both open and closed circles) than under the threshold model (closed circles only). The threshold model is that assumed in Figure 4 of Nowak et al. [13] (*m* = 3, *b*_*0*_ = 0.5, *d*_*0*_ = 0.1, *d* = 0.01, α = 0.1, η = 0.01), with no benefits of working below colony size 3 (two workers). The step model is identical except one worker benefits the queen half as much as two workers do. The data used to make this figure can be found in S1 Dataset.

In addition, the threshold fitness function assumed by Nowak et al. [13] prevents the earliest workers from contributing anything. However, it is easy to envision advantages that would come from having only a single worker [25,32]. To view this effect in isolation, we return to the Nowak et al. [13] decision rule (stay with probability *q*) and to their parameter values given above but allow a single worker to add half the contribution to the queen that two workers add (for both birthrate and death rate) (*m* = 3, *b*_*0*_ = 0.5, *d*_*0*_ = 0.1, *d* = 0.01, α = 0.1, η = 0.01). This simple change (implemented in Equation 1) makes it much easier to evolve eusociality, with *b* = 1.5 or only a 3-fold increase required (Fig. 2) versus 8-fold with the threshold model. This analysis does not resolve what actual fitness functions and decision rules apply in nature, but we note that evolution tends to take the easiest paths available and eschew the difficult ones.

This result appears very close to what is expected under inclusive fitness when r = 1: if two workers increase queen birthrate from 0.5 to 1.5, each raises it by 0.5, exactly the amount that the worker gives up by helping. However, the comparison is not accurate for two reasons. First, this comparison of birthrates neglects the workers’ effect on queen death rate in the model. Second, having gone back to the stay-with probability *q* decision rule, some workers waste their efforts by joining large colonies. In order to compare more closely with inclusive fitness, we altered both of these: the queen death rate is now unchanged by workers, and the stepwise birthrate function is implemented together with the stay-below-colony-size-*w* decision rule. For *w* = 3, eusociality is not favored at b = 1.5 (where inclusive fitness predicts it to be neutral [workers giving up 0.5 and adding 0.5 to the queen]) but is favored to evolve at b = 1.6. It is still possible to argue that eusociality is hard to evolve, depending upon one’s standard for what is hard, but it is considerably easier to evolve than implied by the initial Nowak et al. model and, not surprisingly hard relative to inclusive fitness predictions.

## Discussion

The controversy over the Nowak et al. paper has mostly been conducted at rather abstract levels; different researchers prefer different modeling strategies and may also interpret the evidence differently [13–20,26]. We take a different and more concrete approach by investigating their model for the evolution of eusociality more deeply. If their methods are superior and raise novel insights, we should welcome them and perhaps question our older theories. If instead their methods lead to no novel insights, it undermines the larger claims that the model is used to buttress, specifically that inclusive fitness has not been useful.

We have therefore followed the recommendation of Nowak et al. [13] for modeling social evolution, and in particular eusociality, using deterministic evolutionary dynamics described by ordinary differential equations. However, stimulated by inclusive fitness thinking, we have sought to understand apparent differences between their results compared to previous models. In every case, we find that their rejection of accepted results is incorrect and that in fact the insights known from inclusive fitness theory also emerge using their method.

The claims that relatedness only hastens the spread of eusocial alleles and that relatedness is not causal [13,27] are shown by our models to be false. The proposition could not be tested in the Nowak et al. [13] models because they did not examine any low-relatedness case [15,20]. We have modeled variable relatedness and shown that, under offspring control, high relatedness broadens the range of conditions allowing eusociality to evolve. Relatedness affects not just speed of selection but whether it is favored at all; when relatedness is zero, eusociality does not evolve even with very high benefits (increasing queen birthrate 1,000-fold). This shows that relatedness plays an essential and causal role. Of course, these are not surprising findings because the importance of relatedness was previously well understood from many kinds of models using inclusive fitness [1,7], population genetics [33–35], quantitative genetics [36–38], and game theory [39,40], as well as being supported by much empirical evidence [7,9,41,42].

An alternative interpretation of Nowak et al.’s [13] views is that relatedness is not causal because high relatedness does not always drive the evolution of eusociality. However, this is a rather empty view since no one has ever asserted the contrary and Hamilton’s rule explicitly includes other factors that interact with relatedness. In addition, this view would negate most biological causality of any kind, as no single factor ever completely determines outcomes. Finally, if Nowak et al. agreed that variation in relatedness is an important determinant of eusociality, which is widely regarded as the most important contribution to the topic in 50 years, why did they not say so, instead consistently arguing against its significance? This pattern extends beyond the Nowak et al. [13] paper to Wilson’s earlier and later papers [24,25,27] and to work from Nowak’s group purporting to show new pathways to cooperation [43,44] that in fact depended critically on relatedness and could be interpreted via inclusive fitness [45–47]. Whatever view of causality is taken, it is important to be clear that the Nowak et al. [13] modeling strategy is just like others in showing that higher relatedness is an important factor promoting higher cooperation.

A second claim of Nowak et al. [13], that workers are robots and simply part of the queen’s reproductive success, cannot be made without testing and contrasting queen and worker decision rules. Nowak et al. [13] tested only offspring control models because the decisions are controlled by genes expressed in workers. It is a longstanding result of inclusive fitness theory that parents and offspring are agents with different interests that can be in conflict [28,48]. In particular, in the eusociality context, inclusive fitness predicts that offspring will be selected to help their mothers under a narrower range of conditions than the mothers would favor (eusociality evolves more readily if mothers control the helping of their offspring) (pp. 58–63 of [7]). This follows from differences in relatedness. Workers should gain less from helping less-related kin, but queen inclusive fitness improves if she is less related to the workers who pay the fitness cost.

To examine this question, one must compare selection of offspring agency (genes expressed in the offspring determine whether she becomes a worker) versus maternal agency (genes expressed in the mother determine whether her offspring become workers). We therefore constructed haploid models for maternal control to compare with the results under offspring control. As predicted by inclusive fitness theory, the two cases evolve differently and can be in conflict: mothers favor helping by their offspring under a much broader range of conditions than the offspring themselves favor, except when mothers and offspring are genetically identical (Fig. 1, all circles). And as predicted, when relatedness is low and eusociality is very difficult to evolve under worker control, it is very easy to evolve if the queen has control, because the queen is unrelated to most of the workers who pay the fitness cost. If queens really were in control from the origin of eusociality, and if they could exert that control on unrelated offspring, that would be the easiest path to eusociality. However, this is contradicted by phylogenetic studies showing that relatedness was always high at the various origins of eusociality [41]. In contrast, the standard kin selection model of worker control predicts this observation.

Finally, the claim that eusociality is difficult to evolve [13] is less fundamental than the other two claims and also less wrong because its truth necessarily depends on how one defines “hard to evolve.” Eusociality has evolved a modest number of times and therefore could be viewed as hard to evolve, but their model does make it appear that eusociality is harder to evolve than has been believed. We show that this result hinges on assumptions that are heavily biased towards that conclusion. Little justification was given for why we should accept these particular assumptions. In particular, assumptions are made that imply that many workers waste their efforts. First, their model assumed that offspring stay with probability *q*, independent of any information that might be available about the need for workers. One advantage of inclusive fitness thinking is that it induces researchers to think of workers as agents being selected to get better outcomes (higher inclusive fitness) using whatever information is available to them. One such piece of information is the number of workers already present on the nest. In the threshold fitness model, there is no inclusive fitness gain to be had from staying above that threshold, unless some workers die, so we asked if there was some obvious better decision rule than stay with probability *q*. We therefore tested decision rules that have workers staying when the colony is below a threshold size (not necessarily the same as the fitness threshold) and leaving when the colony is above that size. Not surprisingly, we find that this class of decision rules makes it easier to evolve eusociality, because fewer workers are making wasteful decisions to stay in large colonies. Such a rule seems well within the capabilities of workers. They need not count adults. They simply need to be able to assess some reasonable correlate of the count, something that even microbes do when using quorum sensing to change their behavior. For social insects, the mechanism might involve the degree of comfort with contacting other adults or the hunger demands of offspring.

Similarly, the threshold fitness model assumed by Nowak et al. devalues worker behavior at the other, low, end of colony sizes. In most of their model examples (though not their general model), it was assumed that it was necessary to have two workers to provide any benefit at all to the queen (*m* = 3). That means that the first worker to join a colony provides nothing. However, it is easy to envision situations in which the first worker to join would provide real benefits [32]. The simplest is that at this point one individual can guard the nest while the other forages [25]. Empirical evidence suggests that first helpers do provide benefits [49–54]. If we modify the Nowak et al. threshold model to a step model in which each worker below the threshold adds an additional fixed benefit up to the maximum at colony size *m*, so that the efforts of unjoined first workers are not wasted, eusociality evolves much more easily. Thus, two modifications—the stepped fitness function and the altered worker decision rule—independently make it easier for eusociality to evolve. When we implemented these two rules together so that no workers waste their efforts and assumed workers affect only queen birthrates, eusociality evolved when predicted by inclusive fitness effects on birthrates. We do not know if this is general; the exact correspondence of the two methods may deserve additional study, but our goal here is to address the apparent major discrepancies.

The method advocated by Nowak et al. [13] offers the advantage of specifying parameters like birth and death rates explicitly and following their effects over time while allowing some features, like colony size, to change. We expect that these methods can be used to generate interesting results. However, they are more complex and less intuitive than inclusive fitness thinking, so considerable care is needed to fully understand them. The common thread in the three errors pointed out in this paper is overgeneralization from narrow assumptions or particular parameter values. Relatedness was said to be unimportant even though the models did not vary relatedness. The assertion that workers are not independent agents was made in the absence of models that compared decision rules of different agents. Eusociality was said to be difficult to evolve based on specific and questionable assumptions about the fitness function and offspring decision rules. The more complex the model, the easier it is to be misled by particular results that are not general. In this case, the initial Nowak et al. model [13] missed not just minor details but perhaps the most important generalizations known from the last five decades of theory and empirical study: the importance of relatedness and conflict. Apparent lack of agreement with prior results should have triggered more than a quick rejection of inclusive fitness and kin selection; it should have led to a questioning of why the results were, or seemed to be, different. When examined more closely, models of the type advocated by Nowak et al. [13] do not overturn but instead reaffirm principles of social evolution discovered through inclusive fitness. To have multiple theoretical approaches converging on similar results attests to the robustness of social evolution theory.

## Methods

Our models are all based on the haploid model of Nowak et al. [13]. They modeled the evolution of eusociality with systems of differential equations tracking the number of solitary queens (*x*_*0*_) and eusocial colonies of size *i* (*x*_*i*_). We use a modified notation because our low-relatedness models require us to also keep track of colonies headed by solitary-genotype queens. We therefore let *e*_*i*_ be the number of colonies of size *i* headed by a eusocial queen (that is with *i* – 1 workers) and *s*_*i*_ be the number of colonies of size *i* headed by a solitary queen. With this modified notation, equation set 58 of Nowak et al. [13] can be written as:

$$\begin{array}{l} {\dot{s}}_{1}=(b_{1}\phi -d_{1})s_{1}\\ {\dot{e}}_{1}=\sum_{i=1}^{\infty }b_{i}\phi (1-q)e_{i}-b_{1}\phi qe_{1}-d_{1}e_{1}+\alpha e_{2}\\ {\dot{e}}_{i}=b_{i-1}\phi qe_{i-1}-b_{i}\phi qe_{i}-d_{i}e_{i}-\alpha (i-1)e_{i}+\alpha ie_{i+1}\;\text{for}\;i\text{>1,} \end{array}$$ (1)

where *b*_*i*_ and *d*_*i*_ are the birth and death rates of colonies of size *i*, *q* is the probability that an offspring of a eusocial colony stays as a worker (offspring of solitary colonies never stay), *α* is the worker mortality rate, and *ϕ* is a density-dependent correction factor equal to 1/(1 + *ηX*), with *X* being the total population size including workers and *η* scaling the size of the system.

For specific examples, Nowak et al. [13] usually assumed birthrates and death rates were governed by a simple threshold function: below some threshold colony size *m*, *b*_*i*_ = *b*_*0*_ and *d*_*i*_ = *d*_*0*_ and at or above colony size *m*, *b*_*i*_ = *b* and *d*_*i*_ = *d*. Using two numerical methods (see below), we used Equation 1 to reproduce the results of Figure 4 in Nowak et al. [13] (see S1 Code).

The Nowak et al. models all assumed high and fixed relatedness. We modify their haploid model to incorporate a parameterized mixing step, which allows us to vary the degree of relatedness between queens and workers. The mixing occurs before offspring decide to be workers or reproductive queens. We allowed offspring to move to other mothers, eusocial or solitary, with probability 1 – *r*. Each moving offspring is replaced by a eusocial or a solitary offspring with probabilities *f*_*e*_ and *f*_*s*_, which are simply the proportions of such offspring produced in the population:

$$\begin{array}{l} f_{e}=\sum_{i}b_{i}e_{i}/\sum_{i}b_{i}(s_{i}+e_{i})\\ f_{s}=\sum_{i}b_{i}s_{i}/\sum_{i}b_{i}(s_{i}+e_{i}). \end{array}$$

After mixing, offspring execute their staying rule (leave for solitaries and stay with probability *q* for eusocials). *r* is relatedness to the mother they help because *r* of the time she is identical, and 1 – *r* of the time she is genetically random or unrelated. For this offspring decision model, the equations describing changes in colony types are as follows:

$$\begin{array}{l} {\dot{s}}_{1}=\sum_{i}(b_{i}\phi (1-r)f_{s}e_{i})+\sum_{i}(b_{i}\phi (r+(1-r)f_{s})s_{i})-d_{1}s_{1}+\alpha s_{2}\\ {\dot{s}}_{i}=b_{i-1}\phi (1-r)f_{e}qs_{i-1}-b_{i}\phi (1-r)f_{e}qs_{i}-d_{i}s_{i}-\alpha (i-1)s_{i}+\alpha is_{i+1}\\ {\dot{e}}_{1}=\sum_{i}(b_{i}\phi (r+(1-r)f_{e})(1-q)e_{i})+\sum_{i}(b_{i}\phi (1-r)f_{e}(1-q)s_{i})-b_{1}\phi (r+(1-r)f_{e})qe_{1}-d_{1}e_{1}+\alpha e_{2}\\ {\dot{e}}_{i}=b_{i-1}\phi (r+(1-r)f_{e})qe_{i-1}-b_{i}\phi (r+(1-r)f_{e})qe_{i}-d_{i}e_{i}-\alpha (i-1)e_{i}+\alpha ie_{i+1}. \end{array}$$ (2)

Here *e*_*i*_ and *s*_*i*_ still represent numbers after decision rules are executed and do not reflect numbers in the transient mixing stage. The equations were numerically solved using S3 Code.

For maternal control, we implemented the same offspring mixing model but allowed the mother’s genotype to determine whether the offspring in her colony (some of them resulting from mixing from other colonies) stay and help. Thus, if the queen is eusocial, her (mixed) offspring will become new workers with probability *q* or new queens with probability 1 – *q*. If the queen is solitary, then all offspring will become new queens. The equations now become the following:

$$\begin{array}{l} {\dot{s}}_{1}=b_{1}\phi (r+(1-r)f_{s})s_{1}+\sum_{i}(b_{i}\phi (1-r)f_{s})(1-q)e_{i})-d_{1}s_{1}\\ {\dot{e}}_{1}=\sum_{i}(b_{i}\phi (r+(1-r)f_{e})(1-q)e_{i})+b_{1}\phi (1-r)f_{e}s_{1}-b_{1}\phi qe_{1}-d_{1}e_{1}+\alpha e_{2}\\ {\dot{e}}_{i}=b_{i-1}\phi qe_{i-1}-b_{i}\phi qe_{i}-d_{i}e_{i}-\alpha (i-1)e_{i}+\alpha ie_{i+1}. \end{array}$$ (3)

Note that, unlike the worker model, there are no solitary colonies larger than one (after the transient mixing stage) because a solitary queen always causes her offspring pool to disperse and become reproductive. The equations were numerically solved using S4 Code.

To examine if eusociality is easier to evolve than suggested in Nowak et al. [13], we tested alternative worker decision rule and fitness functions. First, instead of staying with probability *q*, eusocial offspring always stay when colony size *i < w* and always leave when *i ≥ w*. The equations are as follows:

$$\begin{array}{ll} {\dot{s}}_{1}=(\phi b_{1}-d_{1})s_{1} & \\ {\dot{e}}_{1}=\sum_{i=w}^{\infty }\phi b_{i}e_{i}-\phi b_{1}e_{1}-d_{1}e_{1}+\alpha e_{2} & \\ {\dot{e}}_{i}=\phi b_{i-1}e_{i-1}-\phi b_{i}e_{i}-d_{i}e_{i}-\alpha (i-1)e_{i}+\alpha ie_{i+1} & \text{for}\;1<i<w\\ {\dot{e}}_{i}=\phi b_{i-1}e_{i-1}-d_{i}e_{i}-\alpha (i-1)e_{i}+\alpha ie_{i+1} & \text{for}\;i=w\\ {\dot{e}}_{i}=-d_{i}e_{i}-\alpha (i-1)e_{i}+\alpha ie_{i+1} & \text{for}\;i>w. \end{array}$$ (4)

These equations were numerically solved using S5 Code.

We also altered the fitness functions from single thresholds to step functions. Now each added worker adds the same amount, up to the maximum *b* attained at colony size *m*. The maximum gain in both models is the same, but now each worker up to size *m* adds something. We can model this with Equation 1: if *b*_*0*_ is the birthrate of a solitary queen and *b* is the birthrate of a eusocial queen in colony size *m*, then we let the birthrate of queens in smaller colony sizes 1 < *i* < *m* be *b*_*0*_ + (*i −*1)(*b − b*_*0*_)/(*m −*1). Similarly, we let the queen death rate for colony sizes 1 < *i* < *m* be *d*_*0*_ + (*i −*1)(*d − d*_*0*_)/(*m −*1). This implementation of the Nowak et al. model was numerically solved using S2 Code.

To solve the ordinary differential equations, we used two numerical methods. For Equations 1–4, Euler's method was used in R to numerically determine the equilibrium population of the system, using a time step of h = 0.1 and a maximum colony size of n = 50 and terminating when either E or S population/number of individuals was less than ε = 0.1 or after a maximum of 50,000 time steps. Equation 1 was also solved with a first-order numerical procedure with the step size 0.1 implemented in MATLAB. The procedure was started with equal numbers of solitary females and eusocial queens (n = 100) and was terminated when either the solitary or eusocial populations were extinct (defined as less than 0.05) or both the solitary and eusocial populations stabilized at a maximum of 200,000 time steps. Both numerical methods successfully reproduced Figure 4 of Nowak et al. [13].

## Supporting Information

S1 Code R code for the threshold model in Fig. 2. (ZIP)

S2 Code R code for the step model in Fig. 2. (ZIP)

S3 Code R code for worker control under variable relatedness, Fig. 1. (ZIP)

S4 Code R code for queen control under variable relatedness, Fig. 1. (ZIP)

S5 Code MATLAB code for the stay-below-colony-size-w decision rule. (ZIP)

S1 Dataset The data points used in Figs. 1 and 2. (XLSX)
